# Workup and Management of Persistent Neuralgia following Nerve Block

**DOI:** 10.1155/2016/9863492

**Published:** 2016-01-19

**Authors:** Paul David Weyker, Christopher Allen-John Webb, Thoha M. Pham

**Affiliations:** ^1^Columbia University College of Physicians & Surgeons, Columbia University Medical Center, New York, NY, USA; ^2^University of California San Francisco School of Medicine, San Francisco, CA, USA

## Abstract

Neurological injuries following peripheral nerve blocks are a relatively rare yet potentially devastating complication depending on the type of lesion, affected extremity, and duration of symptoms. Medical management continues to be the treatment modality of choice with multimodal nonopioid analgesics as the cornerstone of this therapy. We report the case of a 28-year-old man who developed a clinical common peroneal and lateral sural cutaneous neuropathy following an uncomplicated popliteal sciatic nerve block. Workup with electrodiagnostic studies and magnetic resonance neurography revealed injury to both the femoral and sciatic nerves. Diagnostic studies and potential mechanisms for nerve injury are discussed.

## 1. Introduction

Neurological injuries following peripheral nerve blocks are a relatively rare yet potentially devastating complication depending on the type of lesion, affected extremity, and duration of symptoms. Currently, five types of nerve injury are described that range from neuropraxia (type 1), as evidenced by local myelin loss, to neurotmesis (type 5) demonstrated by complete disruption of the entire nerve and nerve sheath (Table 1) [1]. Given the nature of these injuries and the fact that many procedures are performed in ambulatory surgery centers, it is not uncommon for these complications to go undiagnosed until the first postoperative visit. To facilitate workup and management of these patients, many institutions have initiated a process through which these patients can easily be referred to a physician who specializes in either peripheral nerve injuries or pain management. We present the case of a patient who experienced chronic pain after perioperative femoral and sciatic nerve blocks. The workup and management of peripheral nerve injuries secondary to peripheral nerve blocks will be discussed.

## 2. Case Description

The patient is a 28-year-old man, 175 cm and 82 kg (BMI 26.6) with no past medical history, who tore his left anterior cruciate ligament (ACL) while playing basketball. There were no other associated injuries. When he presented to the orthopedic surgeon he only complained of 2/10 pain on the numeric rating scale (NRS) and instability in his left knee. He denied taking routine daily medications, allergies to medications, illicit drug use, or contributory family history. One month after injury he presented for left knee arthroscopy with ACL reconstruction with hamstring autograft.

Prior to surgery the patient underwent ultrasound guided left sciatic and left femoral nerve blocks. For sedation he received midazolam 2 milligrams intravenously (IV), in divided doses prior to the needle placement. The sciatic nerve block was completed using real time ultrasound with direct visualization of the needle by an anesthesiology resident who was supervised by a skilled regional anesthesiologist. The block was performed immediately distal to the bifurcation of the sciatic nerve into its tibial and common peroneal branches. A Pajunk needle, 21-gauge, length 4-inch (10 centimeters), was used and the needle tip was placed between the tibial and common peroneal nerves. The injectate consisted of 25 milliliters of 0.5% ropivacaine, which was injected in incremental doses with frequent negative aspirations. Perineural, circumferential spread of local anesthetic around the tibial and common peroneal nerves was noted. No paresthesias were noted at the time of injection. The femoral nerve block was completed using a Pajunk needle, 21-gauge, length 2-inch (5 centimeters), using real time ultrasound with direct visualization of the needle. The needle tip was placed underneath the femoral nerve, immediately superficial to the iliopsoas muscle. The injectate consisted of 22 milliliters of 0.5% ropivacaine, which was injected incrementally with frequent negative aspirations. Perineural injection of local anesthetic was noted as the femoral nerve was lifted off the surface of the iliopsoas muscle. No paresthesias were noted at the time of injection. Both nerve blocks provided expected dermatomal sensory anesthesia prior to induction of general anesthesia. No additives such as epinephrine, clonidine, or dexamethasone were added to the ropivacaine injectate.

General anesthesia was induced and maintained with propofol, and a laryngeal mask airway was used. The surgery was uneventful with a total surgical time of approximately 1 hour and 15 minutes. Controlled hypotension [2] with intermittent doses of IV labetalol was used to lower the mean arterial pressure (MAP) in order to mitigate blood loss. No tourniquet was used. Blood loss was noted to be minimal and the patient received 600 milliliters of IV Lactated Ringer's solution. The patient's mean arterial pressure remained consistently between 70 and 80 mmHg for the entire case. He was discharged home the same day. He returned to the orthopedic surgeon for a postoperative visit on postoperative day (POD) 8 and noted good pain control taking only scheduled ibuprofen 400 milligrams by mouth every four hours with an average of 10 mg of hydrocodone (with 325 mg of acetaminophen) by mouth daily. On POD 34, he attended his next follow-up visit with his orthopedic surgeon at which time he noted numbness and pain (NRS 4/10) located across the top and lateral aspects of his left foot in the distribution of the common peroneal (superficial and deep) and the lateral sural cutaneous nerves. He noted that the pain was present immediately after surgery and it progressively worsened over the prior two weeks. He described the pain as burning with intermittent electrical shock sensations and noted no other specific inciting factors. He was diagnosed with an acute post-op neuropathy and prescribed gabapentin 300 milligrams by mouth three times daily. At this time there were no noted motor or sensory deficits on physical exam.

On POD 40, the patient was again seen in the orthopedic clinic for followup due to worsening pain (NRS 7–10/10) that was now impacting his ability to sleep. The pain was now worsened by wearing socks or the brushing of his bed sheets against his left foot. The orthopedic surgeon consulted a pain medicine physician, who recommended he uptitrate his gabapentin to 600 milligrams by mouth three times daily. Additionally, a lidocaine 5% patch was prescribed. Ibuprofen 400 milligrams by mouth every 8 hours was resumed. Two days later he called the orthopedic clinic to say that the lidocaine patch did not work. At this point, amitriptyline 25 milligrams by mouth daily was prescribed and a referral placed for patient to be formally evaluated at the pain management clinic.

He visited the pain management clinic on POD 52. By this time he was taking gabapentin 900 milligrams by mouth three times daily and amitriptyline 25 milligrams at bedtime. The neuropathic pain questionnaire (NPQ) [3, 4] was performed. He noted periods of electrical shock sensations followed by numbness in addition to burning and pinprick sensations in the distribution of the sural and superficial peroneal nerves. The pain improved with cold compress and was worsened by wearing a sock or having anything lightly touching his foot. Brush evoked allodynia was noted on physical exam. Patient was very anxious and deferred pinprick exam. Titration schedules were given to the patient to increase these medications further as pain dictated. An electromyography and nerve conduction study had been completed on POD 45 which revealed a left sciatic mononeuropathy with mild to moderate axon loss in addition to a left femoral neuropathy with mild motor axon loss. A magnetic resonance neurogram (MRN) was ordered at this visit, which showed nonspecific long segmental thickening and increased T2 signal involving the left femoral nerve and extra pelvic left sciatic nerve, which corresponded to the areas where the peripheral nerve blocks were performed. At the time of this visit he did not fit Budapest criteria [5] for a diagnosis of complex regional pain syndrome.

One month later at the pain clinic follow-up visit he noted no benefit with gabapentin despite uptitration to 1200 milligrams by mouth three times daily. He had stopped the amitriptyline due to paradoxical insomnia. He had begun a trial of topiramate starting at 25 milligrams by mouth daily. At this visit it was recommended that he cross-titrate off gabapentin onto pregabalin and that he replace daily ibuprofen with celecoxib. Over the next weeks, he decreased his gabapentin and eventually was transitioned to pregabalin 150 milligrams by mouth twice daily alongside celecoxib 100 milligrams by mouth twice daily. Topiramate had not provided additional relief with intolerable neurologic side effects (dizziness, fatigue, and impaired cognition) and thus was discontinued.

Approximately three months after surgery the patient noted that his pain started to subside. The severe burning sensation in the distribution of the left lateral sural cutaneous nerve and the common peroneal nerves was replaced by pruritis. By four months after surgery his nerve pain was much improved with only occasional bouts of provoked pain, including prolonged periods of sitting and attempts at return to basketball. Rest consistently relieved these flares. He continued to take pregabalin 150 milligrams by mouth twice daily and celecoxib 100 milligrams by mouth twice daily. By six months post-op he was 95% pain-free and was able to wean off pregabalin completely. At last pain clinic visit, he was advised to wean off celecoxib 100 milligrams by mouth as pain allowed and to return to clinic on an as-needed basis. No further followup was needed.

## 3. Discussion

The reported incidence of complications following peripheral nerve blocks is not entirely known and changes depending on timing, definition of neurological injury, and anatomic location of the block. Brull et al. reviewed over 30 studies published between 1995 and 2005. In their review, the estimated risk of neurological complications ranged from 0.03% for supraclavicular blocks up to 2.84% for interscalene blocks [6]. The reported rate for neurological injury following popliteal blocks is 0.24% [6]. In general, the majority (greater than 90%) of neurological injuries related to peripheral nerve blocks will resolve within 4–6 weeks and over 99% will resolve by one year [7, 8]. The main factors affecting recovery include the mechanism of nerve injury and patient comorbidities.

During placement of peripheral nerve blocks, nerves can be injured by three main mechanisms: mechanical injury, chemical injury, and/or ischemic injury [9]. Mechanical injury to the nerve can be secondary to direct needle trauma, compression of the nerve, or stretching of the nerve during positioning. There continues to be inconclusive data regarding nerve damage due to direct fascicular trauma from both blunt and sharp beveled needles. Studies suggest that direct nerve trauma may not necessarily result in nerve damage. It is more likely that the primary cause of neurotoxicity is from the intrafascicular placement of the local anesthetic injectate rather than the direct mechanical trauma. Chemical harm can also occur subsequent to an accurate perineural injection. Chemical injury is related to either the injectate itself or the effects of the injectate on the neurochemical milieu that surrounds the fascicles [9]. This milieu is maintained by both the perineurium, which provides a protective barrier to the fascicles, and the vascular endothelium, which maintains the blood-nerve barrier [9]. Animal models have illustrated that both concentration and duration of local anesthetic and adjuvant exposure are the primary factors contributing to chemical neurotoxicity [10]. Additionally, the volume of injectate can also cause trauma secondary to perineural compression. Compression of the nerve results in elevated intrafascicular pressure, which may exceed the nerve capillary perfusion pressure [9, 11]. Ischemic injury to the nerves can also occur during peripheral nerve blocks when the capillary perfusion pressure is compromised. Regardless of exact etiology, initial injury to the nerves leads to increased spontaneous activity which manifests as paresthesias [9]. In general, it is believed that neurological injury during peripheral nerve blocks is multifactorial and most likely involves different aspects of the mechanisms discussed above.

Workup of peripheral nerve injuries following regional anesthesia involves a thorough history and focused neurological exam. Key variables to determine include patient comorbidities and factors specific to the surgery and anesthetic (Table 2). During the physical exam, it is imperative to differentiate any motor or sensory deficits, the severity of the deficits, the number of nerves involved, and whether the nerve involved is a peripheral nerve versus a nerve plexus versus a nerve root. For patients with pain, differentiating the type of pain, the presence of allodynia or hyperalgesia, and potential pain triggers are important information that can assist in determining an optimal pain regimen.

Studies for evaluating peripheral nerve injuries include both imaging modalities as well as electrodiagnostic studies [12]. Ideally, both studies should be performed during the initial evaluation [12] to provide baseline assessment, with repeat studies guided by patient symptoms and response to treatment [13].

Optimal imaging techniques will vary depending on location of injury and patient related factors (contraindications to magnetic resonance imaging or allergies to commonly used contrast agents). Magnetic resonance imaging (MRI) has become the imaging modality of choice for morphologic evaluation of peripheral nerve injuries. Traditionally, MRI was used to identify vascular compromise or nerve compression (cysts, ganglia, etc.) as a potential cause of peripheral nerve injury [11, 14]. With the advancement of MRI technology and the advent of higher resolution, 3-tesla machines, MRIs are now able to identify signal changes not only within the nerves themselves but also within the surrounding skeletal muscle. While chronic secondary changes within denervated regions may take several weeks to develop and are characterized by atrophy and fatty infiltration, more acute changes such as prolongation of T2 relaxation and increased signal intensity on short tau inversion recovery (STIR) sequences can now be elucidated [14]. A more sophisticated study, magnetic resonance neurography has become increasingly utilized in academic centers for imaging peripheral nerves and may provide clinicians with clues regarding potential mechanisms of nerve injury [15–19].

Nerve conduction studies (NCS) and electromyelography (EMG) are two of the most commonly ordered electrodiagnostic studies to investigate post block neuropathies. They are the only tests that can evaluate the neurophysiologic function of nerves. NCS test the integrity of both motor and sensory nerves by analyzing the speed at which impulses are conducted along the nerves. In general, NCS are able to determine whether or not a nerve lesion is present, the severity of the lesion, to distinguish between a mononeuropathy and a polyneuropathy, and to determine whether axonal loss or demyelination is present [12]. NCS studies across an injured nerve segment will be abnormal immediately. In contrast, EMGs are used to interrogate the integrity of muscle (skeletal muscle and involuntary muscle) innervation and assess the motor unit, motor unit recruitment, muscle insertional activity, and muscle activity at rest [12]. In both of these electrodiagnostic studies, the findings change with time, as the degree of denervation injury cannot be assessed until Wallerian degeneration is complete. Thus it is important to recognize that early EMG studies following the initial injury can provide false negative results [12]. Despite the false negative risk, early post block EMGs can be helpful to differentiate acute injuries from chronic preexisting damage.

Treatment for post block neuropathies remains controversial. In general, the majority of these patients will have resolution of their symptoms in 4–6 weeks with 99% of patients being symptom-free at one year [7, 8]. As with the treatment of many neuropathies or neuralgias, the goal is maintenance of function. Physical therapy remains paramount to prevent muscle atrophy and help mitigate loss of function. In terms of medications, multimodal analgesia with nonopioid analgesics should be the medications of choice (Table 3). When not contraindicated, acetaminophen, nonsteroidal anti-inflammatory drugs (NSAIDs), anticonvulsants, antidepressants, and topical agents such as capsaicin and lidocaine may provide adequate relief. Depending on the degree of neurogenic inflammation seen on imaging, a short course of oral glucocorticoids has been advocated by some. Complimentary medications such as omega-3 fatty acids and vitamin D have also been used by some institutions for treating post block neuropathies. In our patient, the combination of cryotherapy through ice packs in addition to celecoxib and pregabalin provided sufficient pain control to main function both at work and at home despite the injury. It is interesting, however, that while imaging and electrodiagnostic studies demonstrated injury to both the sciatic and femoral nerves, his pain was localized to the sciatic nerve, specifically, the common peroneal and lateral sural cutaneous nerves. Of note, the lateral branch of the sural nerve is derived from the common peroneal nerve while the medial branch arises from the tibial nerve. It is possible that while blocking the sciatic nerve within the popliteal fossa, the needle may have injured the common peroneal nerve or a portion of the local anesthetic was not perineural but rather injected between the fascicles and the epineurium. However this is less likely, as direct trauma to both the sciatic and femoral nerves would be statistically rare. More likely, ropivacaine may have been neurotoxic, despite being without additives and within accepted dose ranges (<3 mg/kg) resulting in femoral and sciatic nerves injuries on both the electrodiagnostic studies and MRN. A chemical injury alongside controlled hypotension may have had an additive effect; however his MAPs remained above 70 mmHg throughout the case. Curiously, the femoral EMG revealed mild axonal loss whereas the sciatic nerve demonstrated moderate axonal loss. It is possible that this difference in degree of injury may have accounted for the clinically evident sciatic neuropathy with a subclinical femoral neuropathy.

## 4. Summary

Regional anesthesia in the form of continuous perineural catheters and single injection nerve blocks is being increasingly utilized for intraoperative anesthesia and postoperative analgesia. As pain physicians, it is not uncommon for our anesthesiology colleagues to seek our expertise in managing these patients. While most of these neuropathies are self-limited and will resolve with time, symptom management is often needed to maintain function and promote surgical recovery and ability to participate in physical therapy. Medical management continues to be the treatment modality of choice with multimodal nonopioid analgesics as the cornerstone of this therapy.

## Figures and Tables

**Table 1:** Seddon-Sunderland Classification of Nerve Injuries [1].

Degree of injury	Type of injury	Characteristics
1	Neuropraxia	Temporary conduction block with preserved axonal continuity. Nerve conducts normally above and below injury, but not across it.

2	Axonotmesis	Continuity of endoneurial sheath, with Wallerian degeneration distal to the lesion. Regenerating axon will restore innervation to original target.

3	Neurotmesis with intact perineurium	Concealed intrafascicular lesion with preserved continuity of fasciculi, but discontinuity of nerve axons.

4	Neurotmesis with intact epineurium	Destruction of fascicular structure with nerve trunk continuity, a strand of disorganized tissue. Requires excision and nerve repair.

5	Complete neurotmesis	Loss of continuity of complete nerve including epineurium, perineurium, endoneurium, and axons.

**Table 2:** Possible mechanisms for perioperative nerve injury [12].

Risk factors	Patient	Anesthetic	Surgical
Preoperative	(i) Preexisting neuropathies (ii) Old age (iii) Chemotherapy (iv) Vascular disease (v) Diabetes mellitus (vi) Multiple sclerosis (vii) Inflammatory or autoimmune disorders		

Intraoperative		(i) Needle trauma (ii) Local anesthetic toxicity (iii) Toxicity related to injectate/adjuvants (iv) Hypotension (v) Improper limb positioning (pressure trauma, nerve stretching)	(i) Tourniquet pressure (ii) Direct trauma during surgery (transection, stretch, compression)

**Table 3:** Nonopioid multimodal analgesic regimen for neuropathic pain.

Supplements/vitamins	(1) Acetyl L-carnitine: 1000 mg–3000 mg per day (2) Alpha lipoid acid: 600 mg–1200 mg per day (3) Fish oil: 1000 mg–2000 mg per day (4) Vitamin C: 1000 mg–1500 mg per day

Nonsteroidal anti-inflammatory drugs (NSAIDs)	(1) Ibuprofen 600 mg–2400 mg per day (2) Naproxen 750 mg–1000 mg per day (3) Diclofenac 150 mg–200 mg per day (4) Meloxicam 7.5 mg–15 mg per day (5) Celecoxib 200 mg–400 mg per day (6) Acetaminophen 2000 mg–4000 mg per day (7) Prednisone taper: 60 mg for 5 days → 40 mg for 5 days → 20 mg for 5 days

Calcium channel antagonists	(1) Gabapentin 1800 mg–3600 mg per day (2) Pregabalin 300 mg–600 mg per day

Sodium channel antagonists	(1) Carbamazepine 300 mg–1200 mg per day (2) Oxcarbazepine 1200 mg–1800 mg per day (3) Topiramate 100 mg–400 mg per day (4) Mexiletine 300 mg–450 mg per day

Topical agents	(1) Lidocaine 5% Ointment: 15 g–20 g per day 5% Patch: 1–3 patches per day. 12 hrs on, 12 hrs off (2) Capsaicin 0.025%–0.075% cream: apply 4 times daily (3) Methyl salicylate/menthol cream (4) Transcutaneous Electrical Nerve Stimulation (TENS) unit

Antidepressants	(1) Tricyclic antidepressants Amitriptyline 75 mg–150 mg per day Nortriptyline 75 mg–150 mg per day Desipramine 75 mg–150 mg per day (2) Selective serotonin-norepinephrine reuptake inhibitors Venlafaxine 75 mg–225 mg per day Duloxetine 60 mg–120 mg per day Desvenlafaxine 50 mg per day Milnacipran 100 mg–200 mg per day
